# MASLD/MASH—New mechanisms and treatments

**DOI:** 10.1097/HC9.0000000000000770

**Published:** 2025-07-14

**Authors:** Vanilla Xin Zhang, Yu-Man Tsui, Irene Oi-Lin Ng

**Affiliations:** 1 Department of Pathology, The University of Hong Kong, Hong Kong; 2 State Key Laboratory of Liver Research, The University of Hong Kong, Hong Kong

## INTRODUCTION

Metabolic dysfunction–associated steatotic liver disease (MASLD) and metabolic dysfunction–associated steatohepatitis (MASH) represent a significant global health concern. MASLD is characterized by a spectrum of chronic liver diseases ranging from hepatic steatosis to progressive fibrosis/cirrhosis and is a major risk factor for HCC.^1^ Recent epidemiological data indicate that MASLD/MASH affects ~38% of adults and between 7% and 14% of children and adolescents worldwide.^1^ In addition to addressing MASLD with efforts in lifestyle modifications, resmetirom, a thyroid hormone receptor beta agonist, was granted approval for the treatment of MASH with liver fibrosis in 2024. However, less than one-third of MASH patients (25.9%–29.9%) respond to resmetirom administration with no worsening of fibrosis,^2^ indicating that more efficient and robust therapeutic options for MASLD/MASH are still in urgent need. To this end, a better understanding of the molecular mechanisms underlying MASLD/MASH is crucial. Here, we highlight a few papers recently published in this Journal on the pathogenesis of MASLD/MASH and their potential therapy.

## UNRAVELING THE COMPLEX PATHOGENESIS OF MASLD/MASH: INSIGHTS FROM RECENT RESEARCH

### Inflammation-mediated mechanisms in MASLD/MASH and fibrosis

It is well established that intrahepatic inflammatory response is a critical component during the pathogenic process of MASLD/MASH, involving multiple immune cells and signaling pathways. Parthasarathy et al reported the pro-inflammatory role of myeloid sphingosine 1-phosphate (S1P) receptor 1 (S1P1) in murine MASH progression. Myeloid cell-specific deletion of S1P1 led to significant attenuation of MASH, accompanied by a decrease in plasma ALT level, reduction of intrahepatic accumulation of pro-inflammatory monocyte-derived macrophages and concomitant increase of restorative macrophage populations. S1P1 deletion also sensitized bone marrow-derived macrophages to lipid-induced apoptosis, which may cause a reduction in the number of monocyte-derived macrophages. These findings uncovered the critical role of macrophage-specific S1P1 signaling in driving MASH inflammation.^3^

In another article of *Hepatology Communication*, Sheng et al^4^ delineated the mechanism of inflammation in MASH and demonstrated the significant upregulation of AMPK-related kinase 5 [also known as NUAK family SNF1-like kinase 1 (NUAK1)] in liver tissues of MASH patients and mice fed with a high-fat diet (HFD). By facilitating caspase 6 activation, NUAK1 promotes pyroptosis, an inflammation-induced programmed cell death. It was found that the inhibition of the NUAK1/caspase 6 axis promoted the interaction between TAK1 and RIPK1, leading to RIPK1 degradation and reduced pyroptosis. This highlights the crucial role of NUAK1–caspase 6–TAK1/RIPK1 axis for controlling pyroptosis and inflammation in MASH.^4^

### The gut–liver axis-mediated mechanisms in MASLD/MASH

The gut–liver axis is increasingly recognized as a pivotal component in the pathogenesis of MASLD/MASH. This axis denotes the complex interplay between the gastrointestinal tract and the liver, facilitated by microbial, immune, and metabolic pathways.^5^ Nguyen et al^5^ reported in an earlier issue that vasoactive intestinal peptide (VIP)-producing neurons suppressed the secretion of hepatoprotective cytokine IL-22 via interaction with VIP receptor 2 (VIPR2) in intestinal type 3 innate lymphoid cells (ILC3s). By utilizing an HFD-induced MASLD mouse model with conditional genetic deletion of Vipr2 in ILC3 [Rorc(t)^Cre^Vipr2^fl/fl^], significantly higher IL-22 production and reduction in liver steatosis were observed in the knockout mice. This attenuation of MASLD was also achieved upon a global inhibition of VIP-producing neurons in mice.^5^ This study has uncovered a novel mechanism of the intestinal neuroimmune circuit during MASLD/MASH development. Modulating VIPergic neuroimmune signaling may present as a novel strategy, in which interventions can be aimed at blocking VIPR2 on ILC3s or inhibiting VIP-producing neurons to enhance intestinal IL-22 levels and reduce steatosis.

### Mitochondrial dysfunction–mediated mechanisms in MASLD/MASH

Mitochondrial dysfunction is another key factor of MASLD/MASH pathogenesis, contributing to perturbations in energy metabolism, impaired fatty acid oxidation and increased production of reactive oxygen species. By using N-acetylgalactosamine (GalNAc)-conjugated siRNA, Guo et al^6^ demonstrated that liver-specific knockdown of mitochondrial amidoxime–reducing component 1 (MTARC1), which was specifically located on the mitochondrial outer membrane for reducing nitrite or N-hydroxylated prodrugs in hepatocytes, protected against diet-induced MASH in multiple MASLD/MASH mouse models (ob/ob, AMLN diet, CDAHFD).^6^ On the other hand, Tsuchiya et al^7^ showed that improvement in mitochondrial function, including upregulation of proteins related to oxidative phosphorylation, the tricarboxylic acid cycle, and fatty acid transport, could ameliorate hepatic steatosis and inhibit the activation of HSCs. These studies implicate a mechanistic basis for mitochondrial functions in MASLD/MASH development.

## EMERGING THERAPEUTIC STRATEGIES AND NOVEL TARGETS FOR MASLD/MASH

### Drug repurposing and nutritional interventions

Drug repurposing, or drug repositioning, is a promising strategy for the development of therapies for MASLD/MASH. Leveraging the known safety and pharmacokinetic profiles of FDA-approved drugs can significantly reduce the time, costs, and risks associated with the drug discovery process. Neurotropin (NTP) has been widely used in China and Japan to treat chronic pains such as neuropathic pain and fibromyalgia. Tsuchiya et al^7^ have investigated the therapeutic effects of NTP on murine MASLD/MASH and found that NTP treatment preserved hepatic mitochondrial functions while inhibiting HSC activation and suppressing lipid accumulation in the liver, suggesting NTP as a beneficial option for MASH treatment, especially given its existing safety profile.

L-Carnitine is an endogenous amino acid derivative known for its role in fatty acid transport into mitochondria for oxidation and subsequent energy production. Clinically, L-carnitine is often prescribed to treat carnitine deficiency. In an earlier issue of this Journal, Lyu et al^8^ explored the utility of L-carnitine in MASH-HCC prevention. Short-term administration of L-carnitine in MASH patients improved the serum ALT/AST levels and NAFLD activity scores by reducing inflammation and improving lipid metabolism gene signatures. Long-term administration of L-carnitine in atherogenic-HFD-induced MASH-HCC mouse model reduced hepatic steatosis, inflammation, fibrosis and suppressed tumorigenesis by repressing MASH-associated transcriptional factor EGR1, thereby reducing activation of the NEDD9/FAK/AKT oncogenic signaling pathway in hepatocytes.^8^

GLP-1 (glucagon-like peptide-1) agonists are therapeutic agents primarily utilized in managing type 2 diabetes, mimicking the action of the endogenous hormone GLP-1, enhancing glucose-dependent insulin secretion and suppressing glucagon release. Xiang et al^9^ demonstrated a novel GLP/FGF dual targeting-agonist, HEC88473, could improve MASLD with well-tolerated safety in a double-blind randomized trial. Upon a 5-week treatment of HEC88473 in 60 patients with type 2 diabetes and MASLD, MRI-proton density fat fraction (MRI-PDFF) was significantly reduced, represented by −47.21% in the treatment group (30.6 mg, *p*=0.0143) versus −15.5% in the placebo group.^9^ This study suggests HEC88473 can be a novel candidate drug for MASLD treatment.

Lanifibranor is a pan-peroxisome proliferator-activated receptor (pan-PPAR) agonist and exerts significant beneficial effects on MASH/fibrosis in a 24-week phase IIb trial by reducing serum inflammatory biomarkers.^10^ Recently, Barb et al investigated the effect of lanifibranor on insulin resistance (IR) and intrahepatic triglyceride (IHTG) content in patients with type 2 diabetes and MASLD.^11^ Upon 24-week lanifibranor treatment, as compared to the placebo group, a significant reduction by ~50% in IHTG level was observed, in which 65% of patients achieved a >30% reduction in IHTG. Hepatic IR improved markedly, as evidenced by reduced fasting hepatic glucose production and hepatic insulin resistance index. Muscle insulin sensitivity increased by 45%, and adipose tissue function was restored, as indicated by a 2.4-fold rise in plasma adiponectin levels. This study provides proof-of-concept for lanifibranor as a therapeutic strategy to reverse IR, reduce hepatic steatosis, and improve cardio-metabolic health in MASLD.

### Targeting specific signaling pathways and hepatocyte-specific genes

To develop targeted interventions for MASLD/MASH prevention and treatment, strategies including inhibiting the NUAK1–caspase 6 axis to reduce pyroptosis,^4^ modulating S1P1 signaling in myeloid cells to decrease pro-inflammatory macrophage accumulation,^3^ and depleting MTARC1 in hepatocytes to attenuate metabolic syndrome,^6^ could potentially be developed by using multiple tools, including adeno-associated virus (AAV)-based gene therapy, lipid-based drug delivery system GalNAc–siRNA, and antibody-based targeted protein degradation technique LYTACs, etc.

## FUTURE DIRECTIONS: TRANSLATING DISCOVERIES INTO CLINICAL SOLUTIONS

**FIGURE 1:**
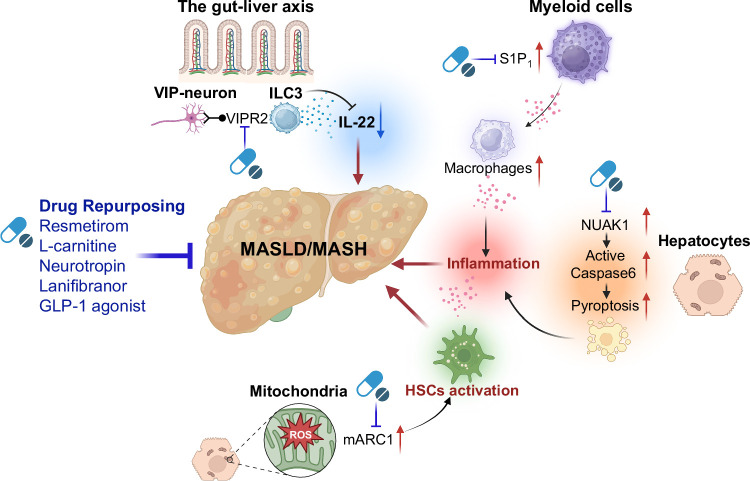
Summary of MASLD/MASH—new mechanisms and treatments. (This figure was generated by Biorender.) Abbreviations: GLP-1, glucagon-like peptide-1; ILC3, intestinal type 3 innate lymphoid cell; MASH, metabolic dysfunction–associated steatohepatitis; MASLD, metabolic dysfunction–associated steatotic liver disease; NUAK1, NUAK family SNF1-like kinase 1; S1P1, sphingosine 1-phosphate receptor 1; VIP, vasoactive intestinal peptide; VIPR2, VIP receptor 2.

The collective findings from the above-mentioned diverse studies significantly advance our understanding of MASLD/MASH and fibrosis pathogenesis and highlight the rich pipelines for potential therapeutic targeting. From modulating specific inflammatory pathways and immune cell behavior to correcting mitochondrial dysfunction, from leveraging neuroimmune interactions to applying targeted genetic interventions, the research landscape for the development of MASLD/MASH therapy is vibrant. Successful repurposing of existing drugs and the exploration of nutritional supplements such as L-carnitine may offer more instant translational possibilities. As our knowledge deepens, a multifaceted therapeutic approach, tailored to individual patient profiles and targeting distinct and complementary mechanisms, will be necessary to effectively combat the MASLD/MASH epidemic. Continuous integration of advanced research methodologies and a strong focus on clinical translation will be paramount in turning these promising discoveries into tangible benefits for patients suffering from MASLD/MASH and fibrosis (Figure 1).
